# Portfolio of learning in clinical training

**DOI:** 10.4102/safp.v67i1.6080

**Published:** 2025-06-04

**Authors:** Louis S. Jenkins

**Affiliations:** 1 Primary Health Care Directorate, Department of Family, Community and Emergency Care, Faculty of Health Sciences, University of Cape Town, Cape Town, South Africa; 2 Department of Family and Emergency Medicine, Western Cape Department of Health, George Hospital, George, South Africa; 3 Department of Family and Emergency Medicine, Faculty of Medicine and Health Sciences, Stellenbosch University, Cape Town, South Africa

**Keywords:** family, medicine, postgraduate, training, portfolio, workplace-based assessment

## Abstract

Workplace-based assessment is increasingly crucial in the postgraduate training of specialists in South Africa, including for family physicians. A portfolio of learning allows a structured, flexible way to present evidence of learning. Portfolios are increasingly digitally based as e-portfolios. Portfolios are used for encouraging self-reflective learning, for transforming learning, and for gathering evidence of skills necessary for future employment. Portfolios support assessment for learning and assessment of learning. This necessitates registrar reflections, supervisor feedback and interaction, and linkages to entrustable professional activities (EPAs). The e-portfolio facilitates triangulation, aggregation and saturation of data points for the various EPAs to support clinical competency committees to make high-stakes evaluations of registrar portfolios. While the initial design and development costs are significant, operational costs become affordable when shared across all training programmes. The portfolio of learning has been a key priority in family medicine for almost 15 years. Initially, a paper-based portfolio was adopted to collect evidence of learning for the national exit-level outcomes. It was converted into an e-portfolio and implemented nationally through the coordination of the South African Academy of Family Physicians. In 2023, the e-portfolio was redesigned to gather evidence of learning for 22 EPAs, and a further revision took place in 2024. A portfolio of learning offers a valuable alternative to traditional assessment methods, allowing for a comprehensive understanding of registrars’ growth over time.

## Introduction

**FIGURE 1:**
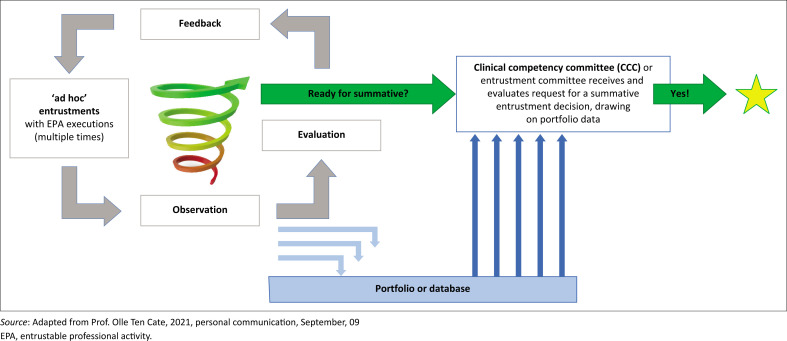
The place of the portfolio in the learning and assessment process.

Workplace-based assessment (WPBA) is increasingly incorporated in postgraduate training of specialists in South Africa.^1^ A portfolio of learning allows a structured, flexible way to present evidence of learning.^2^ Portfolios are increasingly digitally based on electronic portfolios (e-portfolios), with the potential to transform learning and assessment.^3,4^ In WPBA, portfolios are used for encouraging self-reflective autonomous learning, for transforming teaching and learning (from teacher to learner-centred), and for gathering evidence of the skills necessary for future employment.^5^ Portfolios should not only be collections of artefacts but should also contain a purposeful aggregation of evidence of a registrar’s learning and abilities and create opportunities for personal development through registrar reflection and supervisor feedback.^6^ In programmatic assessments, the many low-stakes assessment episodes (observations, reflections, learning conversations and longitudinal assessments) represent multiple data points.^7^ Figure 1 explains how these data points constitute ‘ad hoc’ entrustment decisions by supervisors, captured in a portfolio of learning, for an eventual high-stakes assessment.

**FIGURE 2:**
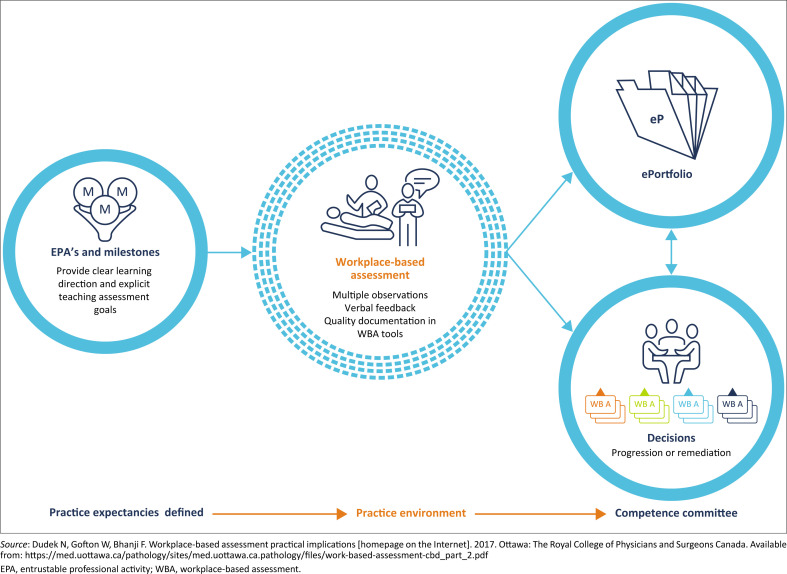
Role of the portfolio in workplace-based assessment.

In South Africa, learning portfolios are one of the acceptable forms of continuing professional development by the Health Professions Council guidelines. Figure 2 represents another view of understanding the role of portfolios of learning in WPBA.

## Benefits of using a portfolio of learning

### Continuous assessment

Instead of relying solely on a single test or examination, portfolios provide a comprehensive picture of registrars’ growth over time.

### Personalised learning

Portfolios can be tailored to each registrar’s unique learning needs. Registrars can showcase their strengths and interests through a diverse range of artefacts, including written assignments, observations, presentations and reflections.

### Formative feedback

Portfolios encourage ongoing feedback and dialogue between supervisors and registrars. Instead of receiving a final grade at the end of a course, registrars receive regular feedback throughout their training. This enables registrars to reflect on their progress, make improvements and set goals in their next learning plan. Keeping a portfolio at hand, planning for learning, regularly writing a small reflection, being observed by a supervisor or peer, with feedback, at least once a month, and having a learning conversation once or twice a month, is a powerful way to manage registrars’ own learning and influence their development and work performance positively.

### Reflection and metacognition

Portfolios promote reflective thinking and metacognitive skills. Through the process of curating and organising their work, registrars develop a deeper understanding of their own learning process, strengths, and areas for improvement.

## Challenges of using a portfolio of learning

### Time and organisation

Implementing a portfolio assessment system requires careful planning and organisation. Without proper time management and guidance, portfolios can become overwhelming for both supervisors and registrars. Structuring and integrating individual learning by noticing and reflecting on experiences in the workplace, and changing practice accordingly, requires a mind shift, which takes time and needs support from an available and skilled supervisor, in a safe learning environment. The South African clinical work environment is a harsh place with limited supervision and a heavy burden of sick patients.^8^

### Subjectivity

Assessing portfolios involves a certain degree of subjectivity. Different supervisors may interpret the artefacts and reflections differently, leading to variations in assessment outcomes. To address this challenge, it is essential to establish clear assessment criteria that guide the evaluation process. With the introduction of entrustable professional activities (EPAs) in medical education, a standardised 5-point entrustability scale is applied throughout.^9^

### Authenticity

The authenticity of a portfolio can be a concern, as there is potential for registrars to include only work that reflects well and leave out areas of concern or difficulty in their clinical practice. Having many ‘data points’ of various clinical learning experiences with multiple supervisors over time greatly enhances the authenticity of assessments. Narrative registrar reflections and supervisor feedback add great value to quantitative data points and entrustability scales.

### Cost

Developing and maintaining an e-portfolio assessment process could be prohibitive for many South African universities and similar educational institutions in sub-Saharan Africa.

The South African portfolio of learning for postgraduate family medicine trainingIt is a requirement for the national exit examination of the Colleges of Family Physicians of South Africa to submit a satisfactory portfolio that shows evidence of learning over 3 years in an accredited training programme. A national portfolio has been developed for family medicine training and WPBA in South Africa, with consensus on the contents and the main assessment tools and is available on the Colleges of Medicine of South Africa (CMSA) website.^10^ The portfolio was initially paper-based and contained in a lever arch file. Subsequently, the paper-based portfolio format was converted to an electronic format, accessible on a computer, tablet, or cell phone, as an e-portfolio.^11^ After the e-portfolio was implemented across the country, the discipline of family medicine reached a national consensus on 22 EPAs, which necessitated a redesign of the e-portfolio (Table 1).^12,13,14^ The annual portfolio assessments were replaced by local clinical competency committees that make a summative entrustment decision for each EPA twice a year based on the evidence provided.^15^

**TABLE 1:** Content of the e-portfolio redesigned for workplace-based assessment.

Number	Element in the portfolio	Description
1	Shared folders	A collection of 21 documents that introduce the portfolio provides a preamble to the EPAs and the 22 EPAs and provides background reading on WPBA.
2	Learning plans, reflections on allocations and supervisor’s assessments	Choose 3–4 EPAs for each learning plan and align these with the reflections and periodic assessments. Allocations can be within the generalist setting (e.g. wards in a district hospital) or to specialist settings (e.g. disciplines in a regional or tertiary hospital).
3	Educational meetings with the supervisor	Registrars meet regularly with their supervisors for learning conversations. These are often group meetings and can be used for clinical governance activities.
	Entrustment-based discussions	Individual case-based or scenario-based discussions
4	Observations of the registrar by the supervisor	A variety of tools are available: Mini-Clinical Evaluation Exercise (Mini-CEX) (for the consultation) Communication skills observation tool Direct observation of procedural skills (DOPS) (for procedures) Caesarean section observation tool Anaesthetic observation tool Teaching or presentation assessment tool (for group teaching events) One-minute preceptor tool (for one-on-one teaching)
5	Multisource feedback	Multisource feedback on performance and entrustability once a year from 10–20 people who report to, work alongside or supervise the registrar.
6	Written assignments	Assignments from their academic programmes that demonstrate application of theory in the workplace (e.g. quality improvement projects and community-oriented primary care projects).
7	Logbook of clinical skills	More than 245 clinical skills are categorised in a mutually exclusive approach into the 22 EPAs. The logbooks record exposure to or experience with each skill over the training programme.
8	Emergency medicine certificate(s)	Certificates from emergency medicine courses (e.g. advanced trauma and life support, advanced paediatric life support or advanced cardiac life support).
9	Other courses, workshops, conferences	Other evidence of pertinent learning from courses, workshops or conferences
10	Clinical competency committee form	An interim and final form allows the competency committee to record a summative entrustment decision for each EPA based on the quantitative and qualitative data in the portfolio.

WPBA, workplace-based assessment; EPA, entrustable professional activity.

The portfolio is not merely a repository for forms that assess learning but support the educational principle of ‘assessment for learning’.^6^ Each data point is an opportunity for constructive feedback or reflection that stimulates further learning. Feedback from the supervisor to the registrar is core to the learning process and critical to be captured in the portfolio. Effective supervisor feedback is dependent on many factors, including established registrar–supervisor relationships, a collaborative learning agenda, a safe space to invite registrar self-reflection (about a skill or action), enhancing self-efficacy and establishing action plans for growth.^16^ Therefore, supervisors must be skilful in providing and recording feedback. Faculty development is needed to enable this. Feedback is structured into three questions: what was done well, what could be done better, and what the learner needs to do next (an action plan). In addition, traditional scores in the various forms have been replaced with ad hoc entrustment decisions. The clinical supervisor records an ad hoc entrustment decision for the registrar for a data point on a scale from 1 to 5, as follows:

- Can observe only;
- Direct supervision (the supervisor must be next to the registrar);
- Indirect supervision (the supervisor must be available in the facility);
- Distant supervision (the supervisor can be available off-site at a distance);
- Supervising others (no supervision is needed).

This approach to the design and use of the portfolio emphasises the importance of trust between registrars and supervisors, the relevance of the clinical context, and the creation of a learning culture that facilitates WPBA.^17,18^

Three key principles of WPBA include triangulation, aggregation and saturation. Triangulation from various data points from different assessors in different settings supports an entrustment decision on each EPA. Aggregation requires that the data points are designed in such a way that they aggregate to specific EPAs. Saturation is achieved when enough data points support an entrustment decision on the EPA. Subsequently, the portfolio supports ‘assessment of learning’. These data points serve as evidence in making summative entrustment decisions for each EPA to ascertain readiness for the national exit exams.

An important part of a portfolio is a personal learning plan, which is prepared before every clinical workplace allocation. Registrars review their prior learning, expected training outcomes, the national list of procedural skills and the clinical service expectations and draft a learning plan. This is done in conversation with their supervisor, who guides them in their learning journey for that specific clinical allocation. At the end of every clinical allocation, registrars reflect on their learning experience. An end-of-allocation assessment by the registrar’s supervisor gives an indication of how the supervisor views the registrar’s performance. It is more important to receive constructive feedback from the supervisor or trainer regarding registrar performance than just receiving a grade. This feedback should be early, specific, sensitive, direct and allow for improved future performance.

Implicit in the learning journey are various learning conversations, or educational meetings, between the registrar and various supervisors. These can be a combination of individual or group discussions. They should capture the thoughts of the registrar and supervisor around a learning event and indicate what would be done differently (better) in future, if confronted with a similar situation. More recently, the entrustment-based discussion was introduced as a type of patient- or scenario-based discussion, with the added question of ‘What if things are different for a similar case in future?’, assessing predictive competence.^19^ While it is best to schedule a regular weekly or two-weekly meeting between registrar and supervisor, registrars will also notice daily opportunities for learning in the workplace. Tools to assist the registrar and supervisor (e.g. how to conduct case-based discussions, clinical question analysis, chart-stimulated recall or a significant event analysis) are available in the portfolio.

Direct and indirect (by video or audio) observations by a supervisor or a senior peer of the registrar’s performance are valid ways to assess performance and to give immediate registrar feedback. Various tools in the portfolio assist with this (e.g. the mini-Clinical Evaluation Exercise [mini-CEX]), direct observation of procedural skills (DOPS), and observation of consultation skills or a teaching session. Written assignments are required to demonstrate the ability to engage with critical issues and principles of family medicine in the real-world environment and to learn from them. For example, this includes demonstrating the ability to resolve ethical dilemmas, plan for community-orientated primary care, critically appraise and apply research evidence, or perform a quality improvement cycle.

Over 245 clinical skills required by a specialist family physician in the South African context have been defined, and the incremental competency to perform these skills is captured in a logbook of procedures, embedded in the portfolio. The goal is not a compliance exercise of ticking as many boxes as possible to get through a list of logbook skills and show the number of skills done. It is more important to provide evidence of learning that will convince a faculty competency committee that the registrar can be trusted with a certain set of skills, encapsulated in an EPA, to be safe and competent when engaging patients in the workplace.^12^

E-portfolios have advantages over paper-based portfolios. Learning can be conveniently captured on a cellular phone or computer. While it remains a challenge for registrars to reflect deeply and for supervisors to provide meaningful feedback, these are more conveniently entered electronically and are clear to read, versus handwritten notes on paper.^5^ A summary of all feedback is easier to visualise electronically, giving a picture of training and development over time. Registrar and supervisor portfolio (and by inference training) engagement are easier to measure electronically.

## Conclusion

A portfolio of learning offers a valuable alternative to traditional assessment methods, allowing educators to gain a comprehensive understanding of registrars’ abilities and growth over time. By embracing the benefits of programmatic assessment, personalised learning, formative feedback and reflection, portfolios can become powerful tools for both registrars and supervisors. While challenges related to time management, subjectivity and authenticity exist, these can be addressed through careful planning, clear criteria, scaffolded support and ongoing faculty development.
